# ERC accumulation depletes Sir2 from rDNA and induces cellular senescence by rDNA destabilization

**DOI:** 10.1093/nar/gkaf923

**Published:** 2025-10-29

**Authors:** Yoshio Yamamuro, Yuta Uneme, Sihan Li, Toshifumi Inada, Takehiko Kobayashi

**Affiliations:** Laboratory of Genome Regeneration, Institute for Quantitative Biosciences (IQB), The University of Tokyo, 1-1-1 Yayoi, Bunkyo-ku, Tokyo 113-0032, Japan; Department of Biological Sciences, Graduate School of Science, The University of Tokyo, 7-3-1 Hongo, Bunkyo-ku, Tokyo 113-0033, Japan; Laboratory of Genome Regeneration, Institute for Quantitative Biosciences (IQB), The University of Tokyo, 1-1-1 Yayoi, Bunkyo-ku, Tokyo 113-0032, Japan; Department of Biological Sciences, Graduate School of Science, The University of Tokyo, 7-3-1 Hongo, Bunkyo-ku, Tokyo 113-0033, Japan; Division of RNA and Gene Regulation, The Institute of Medical Science, The University of Tokyo, 4-6-1, Shirokanedai, Minato-ku, Tokyo 108-8639, Japan; Division of RNA and Gene Regulation, The Institute of Medical Science, The University of Tokyo, 4-6-1, Shirokanedai, Minato-ku, Tokyo 108-8639, Japan; Laboratory of Genome Regeneration, Institute for Quantitative Biosciences (IQB), The University of Tokyo, 1-1-1 Yayoi, Bunkyo-ku, Tokyo 113-0032, Japan; Department of Biological Sciences, Graduate School of Science, The University of Tokyo, 7-3-1 Hongo, Bunkyo-ku, Tokyo 113-0033, Japan; Collaborative Research Institute for Innovative Microbiology, The University of Tokyo, 1-1-1 Yayoi, Bunkyo-ku, Tokyo 113-0032, Japan

## Abstract

Genome instability is a major factor contributing to cellular senescence. The rRNA gene (rDNA), a repetitive sequence array, is a highly unstable region of the genome. In budding yeast, this instability induces senescence and shortens the lifespan. While the importance of rDNA stability in the aging process is well recognized, the mechanism driving rDNA instability in old cells remains unclear. Using effective methods to isolate old cells in budding yeast, our observations suggest that non-coding RNA transcription from the bidirectional promoter E-pro increases by acetylation of histones H3K14 and H4K16, thereby triggering rDNA instability in old cells. Depletion of Gcn5, the enzyme responsible for H3K14 acetylation, reduced E-pro transcription and mitigated rDNA instability in old cells. Contrary to previous studies, the level of Sir2, a deacetylase for H3K14 and H4K16, does not decline with aging. However, acetylation levels at the E-pro region increase, promoting non-coding RNA transcription and rDNA instability in old cells. This phenomenon appears to be driven by Sir2 depletion from chromosomal rDNA, caused by the accumulation of extrachromosomal rDNA circles (ERCs). We propose a new model of cellular senescence in budding yeast, driven by Sir2 depletion and rDNA instability.

## Graphical Abstract

**Graphical Abstract:**
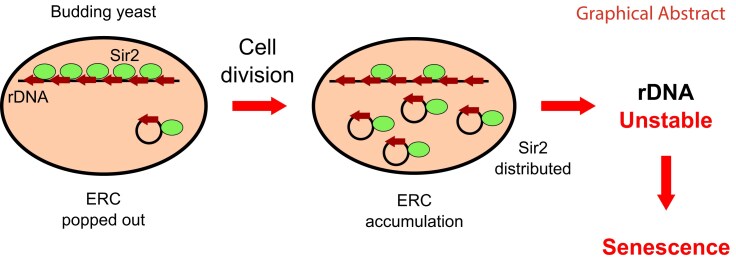

## Introduction

**Figure 1.**
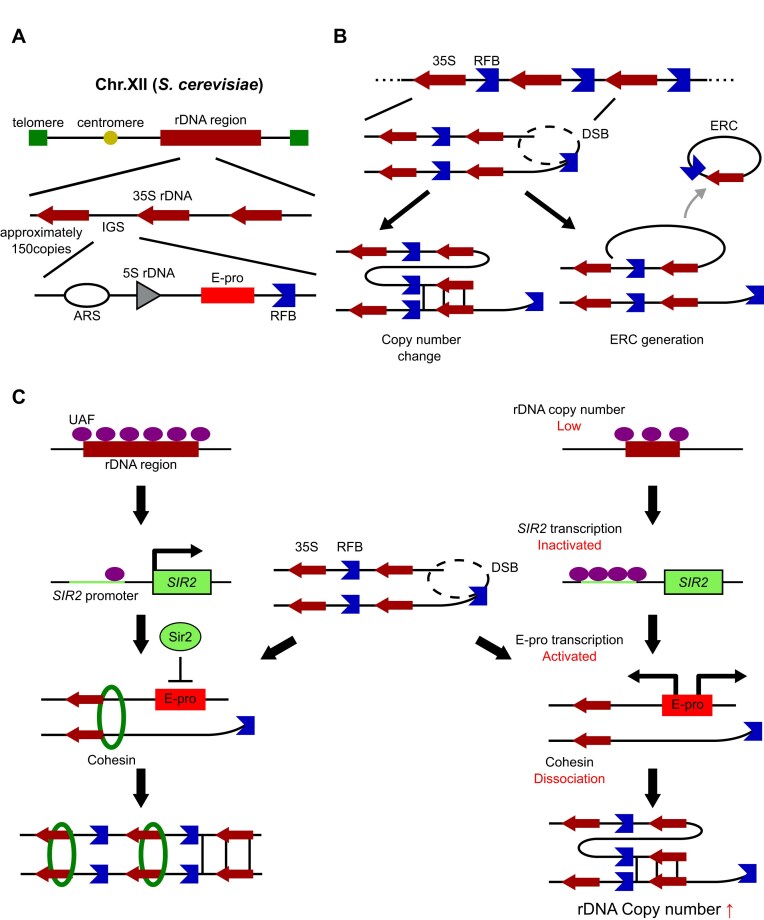
Mechanism of rDNA recombination in budding yeast. (**A**) Structure of rDNA in budding yeast. The rDNA is located on chromosome XII and forms a tandem array (∼150 copies). Each repeat unit contains the 35S and 5S rRNA genes (35S and 5S rDNA). The intergenic spacer (IGS) includes several elements involved in recombination, such as an autonomously replicating sequence (ARS; the replication origin), E-pro (a non-coding promoter), and the replication fork barrier (RFB). (**B**) Mechanism of rDNA recombination leading to copy number changes. Recombination events within the rDNA regions can alter copy numbers, contributing to genomic instability. (**C**) Copy number regulation system. When the rDNA copy number is reduced, upstream activating factors (UAFs), which normally enhance 35S rDNA transcription, are released from their binding sites in the rDNA. The released UAFs bind to the SIR2 promoter, repressing its transcription and thereby reducing Sir2 levels. As Sir2 levels decline, E-pro activity, which is normally repressed by Sir2, becomes activated. This activation promotes unequal sister chromatid recombination, leading to rDNA amplification to restore the copy number.

Genome instability with aging is considered a hallmark of senescence [1]. Previous studies have shown that DNA lesions and mutations increase, while DNA repair efficiency decreases, with aging [2], suggesting a link between genome instability and senescence. The rRNA gene (rDNA) is a highly repetitive region in the eukaryotic genome. Due to its repetitive structure and high transcriptional activity, rDNA is one of the most unstable regions in the genome [3]. The rDNA in budding yeast has been extensively studied [4, 5]. In budding yeast, rDNA consists of ∼150 tandem repeats located on chromosome XII (chr. XII) [6–8], with a replication fork barrier (RFB) site at the end of the gene (Fig. 1A) [9]. DNA double-strand breaks (DSBs) frequently occur at the RFB during the S phase of the cell cycle [10, 11]. These breaks are repaired through recombination [12]. However, due to the tandem repeat structure of the rDNA, repair may occur using an unequally positioned copy, resulting in fluctuations in copy number (Fig. 1B). This frequent fluctuation in rDNA copy number is referred to as rDNA instability, which is considered a cause of cellular senescence [13].

E-pro, located in the intergenic space (IGS) of rDNA, is a bidirectional promoter that transcribes non-coding RNA [14] (Fig. 1A). Activation of transcription at E-pro leads to the dissociation of cohesin, a protein complex that holds sister chromatids together, across the rDNA array, thereby causing rDNA instability [15]. This instability arises from an increase in unequal sister chromatid recombination during the repair of double-strand breaks (DSBs) at the RFB [16, 17].

Sir2 is one of the key factors for E-pro transcription regulation. In *sir2* mutants, E-pro transcription is significantly up-regulated [17], resulting in rDNA destabilization [18]. Consequently, the lifespan of *sir2* mutants is reduced to approximately half that of the wild-type strain level [19]. Sir2 acts as a regulator of rDNA copy number [20] (Fig. 1C). When the rDNA copy number is reduced, upstream activation factors (UAFs) that facilitate 35S rDNA transcription lose their binding sites in the rDNA and are released. These UAFs subsequently relocate to the *SIR2* promoter, repressing its transcription. As a result, Sir2 levels decrease, leading to the activation of the E-pro element, which promotes recombination to restore the rDNA copy number. Once the copy number is restored, UAFs return to the rDNA from the *SIR2* promoter, increasing Sir2 levels and inhibiting further amplification by repressing recombination through E-pro regulation.

Sir2 is an nicotinamide adenine dinucleotide (NAD^+^)-dependent histone deacetylase that silences gene expression [21, 22]. Sir2 belongs to the sirtuin family, which includes its mammalian homologs. Sirtuins also regulate lifespan in mice [23]. In budding yeast, Sir2 silences mating-type loci [24], telomeres [25, 26], and the rDNA region. Sir2 deacetylates lysine 9 and 14 of histone H3, and particularly lysine 16 of histone H4. All of these deacetylation events are critical for silencing [21, 27, 28], and they occur at E-pro in the rDNA [29–31]. In relation to aging, both Sir2 and NAD^+^ levels decline with age [32, 33]. Isonicotinamide (INAM) enhances Sir2 activity by increasing NAD^+^ concentration [34] and counteracts nicotinamide (NAM), an inhibitor of Sir2 [35]. Adding INAM to yeast cultures has been shown to extend their lifespan [34].

rDNA instability is often associated with the production of extrachromosomal rDNA circles (ERCs). ERCs consist of one or more rDNA copies and contain an autonomously replicating sequence (ARS) (Fig. 1A). These ERCs are asymmetrically inherited, with a bias toward the mother cell, leading to their exponential accumulation. This accumulation is thought to contribute to cellular senescence [36]. ERCs have been identified as a cause of genome-level mis-segregation (GLM) and the senescence entry point (SEP), which occur ∼5 generations before cell death [37–39]. However, lifespan shortening associated with rDNA instability has also been observed in ERC-less mutants, suggesting that pathways deeply linked to genome instability itself play a central role in cellular senescence [40].

The relationship between rDNA instability and Sir2 has been established, but the mechanism driving rDNA instability with aging remains unclear, as the specific changes occurring in old cells are not well understood. In this study, we analyzed rDNA instability in old cells and observed that Sir2 on genomic rDNA appears to be depleted due to the accumulation of ERCs. This depletion is suggested to lead to histone acetylation at E-pro, activating transcription and causing rDNA instability. These findings suggest a new model of cellular senescence driven by rDNA instability.

## Materials and methods

### Yeast strains and cultivation

Yeast strains and plasmids used in this study are listed in Supplementary Tables S1 and S2. YPDA medium (1% w/v yeast extract, 2% w/v peptone, 2% w/v glucose, 2% w/v agar, and 0.4% w/v adenine) or SC medium [0.67% w/v Yeast Nitrogen Base without amino acids, 2% w/v glucose (or galactose in Fig. 3B,C), 20 mg/l l-arginine HCl, 60 mg/l l-tyrosine, 80 mg/l l-isoleucine, 50 mg/l l-phenylalanine, 100 mg/l l-glutamic acid, 100 mg/l l-aspartic acid, 150 mg/l l-valine, 200 mg/l l-threonine, 400 mg/l l-serine, 20 mg/l uracil, 40 mg/l adenine sulfate, 60 mg/l l-leucine, 40 mg/l l-tryptophan, 20 mg/l l-histidine HCl, 20 mg/l l-methionine, and 120 mg/l l-lysine HCl, with the appropriate amino acid removed] was used for cultivation unless otherwise specified. For plate media, 2% agar was added. The strains were streaked out, and independent colonies were transferred into liquid media and incubated at 30°C. Before using strains NOY408-1b YEplac181 and NOY408-1b YEplac181-IGS, they were incubated for ∼30 generations in medium with the appropriate sugar. INAM treatment was performed by adding INAM (Sigma-Aldrich) to the medium at a final concentration of 25 mM.

### Sorting of old cells

Old cells were sorted as described previously [41] with modifications. Logarithmic phase cells were collected by centrifugation and washed with phosphate-buffered saline (PBS). They were suspended in 100 μl of PBS (for 10^7^ cells), and 1.3 mg of sulfo-NHS-LC-biotin (Thermo Fisher Scientific) in 40 μl of PBS was added, followed by incubation at room temperature for 15 min. After incubation, the cells were collected, washed with PBS, suspended in 100 μl of PBS, transferred into 2.5% glucose YPDA medium (1% w/v yeast extract, 2% w/v peptone, 2.5% w/v glucose, 2% w/v agar, and 0.4 % w/v adenine), and incubated. After the cells were allowed to divide seven times, they were collected by centrifugation and suspended in 4 ml of PBS. A 40 μl aliquot of streptavidin-coated magnetic beads (Thermo Fisher Scientific) that were washed with PBS twice was added to the yeast suspension, and the mixture was incubated at room temperature for 15 min, followed by eight washes with PBS. Young cells were stored after the first wash. To measure bud scars, the cells were suspended in 4% (w/v) paraformaldehyde, incubated at room temperature for 5 min, washed three times with PBS, suspended in PBS, and stained with Calcofluor white (Sigma) (final 100 mg/μl) for 5 min. After two additional PBS washes, the cells were visualized by fluorescent microscopy.

### Genomic DNA preparation

For pulsed-field gel electrophoresis (PFGE) and ERC assay, genomic DNA was prepared in low melting temperature agarose plugs as described previously [12, 42]. Briefly, stationary phase cells were collected and resuspended in 50 mM EDTA (pH 7.5) at a concentration of 33 μl per 5 × 10^7^ cells. The cell suspension was incubated at 40°C and mixed with 66 μl of solution 1 [0.83% w/v low-melting-point agarose SeaPlaque GTG (Lonza), 170 mM sorbitol, 17 mM sodium citrate, 10 mM EDTA pH 7.5, 0.85% v/v β-mercaptoethanol, and 0.17 mg/ml Zymolyase 100T (Nacalai)]. The mixture was poured into a plug mold (Bio-RAD) and placed at 4°C for 20 min to solidify. The plugs were then transferred into solution 2 [450 mM EDTA (pH 7.5), 10 mM Tris–HCl (pH 7.5), 7.5% v/v β-mercaptoethanol, and 10 μg/ml RNase A (Macherey-Nagel)] and incubated at 37°C for 1 h. Subsequently, they were incubated overnight at 50°C in solution 3 [250 mM EDTA (pH 7.5), 10 mM Tris–HCl (pH 7.5), 1% w/v sodium dodecyl sulfate (SDS), and 1 mg/ml proteinase K (Nacalai)]. The plugs were washed four times for 15 min with 50 mM EDTA (pH 7.5).

### Pulsed-field gel electrophoresis

PFGE was performed as described previously [12, 42]. Briefly, one-third of a plug or *Hansenula wingei* chromosomal DNA markers (Bio-Rad) was placed on a comb tooth. The comb was set into the gel tray, and 1% agarose solution (Pulsed Field Certified Agarose, Bio-Rad) in 0.5× TBE [44.5 mM Tris base, 44.5 mM boric acid, and 1 mM EDTA (pH 8.0)] was poured. PFGE was run on a Bio-Rad CHEF DR-III system in 2.2 l of 0.5× TBE under the following conditions: 3.0 V/cm for 68 h at 14°C, 120° included angle, initial switch time of 300 s, and final switch time of 900 s. After electrophoresis, DNA was stained with 0.5 μg/ml ethidium bromide (EtBr) for 30 min, and washed twice with water for 30 min each before being photographed. The stability of rDNA was assessed using Fiji (ver.2.16.0) by extracting the signal from chr. IV, measuring its width, and then extracting a segment of the same width centred on the signal peak on chr. XII. The area of the peaks was calculated for chr. XII and chr. IV in the density profile plot, using the endpoints of the extracted segment as the base. The ratio of these areas (chr. XII/chr. IV) was then determined.

### Extrachromosomal rDNA circle assay

ERC assay was performed as described previously [42, 43]. Half of a plug was placed on a comb tooth. The comb was set into the gel tray (15 × 25 cm), and 300 ml of 0.4% agarose (LABTAS + Agarose Powder, LABTAS+) in 1× TAE [40 mM Tris base, 20 mM acetic acid, and 1 mM EDTA (pH 8.0)] was poured into the tray. A 500 ng aliquot of lambda HindIII DNA marker was applied to an empty lane. The electrophoresis was performed using a Sub-cell GT electrophoresis system (Bio-Rad) in 1.5 l of 1× TAE at 1.0 V/cm for 48 h at 4°C with buffer circulation. The buffer was changed after 24 h. DNA was stained with 0.5 μg/ml EtBr for 30 min before being photographed.

### Southern blot analysis

#### DNA transfer

Southern blot was performed using gels from the PFGE or ERC assay as described previously [44] with modifications. When Hybond-XL (GE Healthcare) was used, the gels were incubated in 500 ml of 0.25 N HCl for 20 min, followed by 500 ml of 0.5 N NaOH, 1.5 M NaCl for 20 min. DNA was transferred to the membrane with 0.25 N NaOH, 1.5 M NaCl overnight. The membrane was soaked in 100 ml of 0.4 N NaOH for 10 min, followed by 100 ml of 2× SSC for 10 min. When Hybond-N+ was used, the gels were incubated in 500 ml of 0.25 N HCl for 20 min, 500 ml of 0.5 N NaOH, 1.5 M NaCl for 20 min, and 500 ml of 0.5 M Tris–HCl (pH 7.5), 1.5 M NaCl for 20 min. DNA was transferred to the membrane with 10× SSC overnight. DNA was fixed by UV cross-linking at 120 000 μJ/cm^2^ using a Stratalinker (Stratagene, Model 1800), and the membrane was soaked in 5× SSC for 10 min.

#### DNA probe preparation

Probes were prepared as described previously [12, 42] with slight modifications. Double-stranded DNA fragments were amplified by PCR. The first PCR was performed using NOY408-1b as the template. The primers used for this PCR are listed in Supplementary Table S4. The product was gel-purified and used as the template for a second round of PCR. The PCR product was gel-purified, and 50 ng was used for random priming reactions in the presence of the radiolabeled nucleotide, [α-^32^P]dCTP (3000 Ci/mmol, 10 mCi/ml, PerkinElmer), using the Random Primer DNA Labeling Kit Ver.2 (TaKaRa). Unincorporated nucleotides were removed using ProbeQuant G-50 Micro Columns (GE Healthcare). The radiolabeled probes were heat-denatured for 3 min at 100°C immediately prior to hybridization to the membrane.

#### Hybridization

Southern hybridization was performed as described previously [12, 42] with slight modifications. The membrane was pre-wetted with 0.5 M phosphate buffer (pH 7.2) and pre-hybridized for 30 min at 65°C in 25 ml of hybridization buffer [1% w/v bovine serum albumin (BSA), 0.5 M phosphate buffer (pH 7.2), 7% w/v SDS, 1 mM EDTA (pH 8.0)]. After discarding the buffer, the membrane was hybridized with 25 ml of hybridization buffer containing the heat-denatured probe overnight at 65°C. The membrane was washed four times for 15 min each at 65°C with wash buffer [40 mM phosphate buffer (pH 7.2), 1% w/v SDS, 1 mM EDTA (pH 8.0)] and exposed to a phosphor screen. ERC signals were normalized to genomic rDNA signals in the quantification (ERC/genomic rDNA).

### RNA extraction

Logarithmic phase cells were collected, and RNA was extracted using either the RNeasy Mini Kit (QIAGEN) or the NucleoSpin RNA (Macherey Nagel). For quantitative PCR (qPCR), reverse transcription and genome DNA removal were performed using ReverTra Ace® qPCR RT Master Mix with gDNA Remover (TOYOBO).

### DNA extraction

DNA was extracted as described previously [45] with slight modification in Fig. 3D. Stationary phase cells were collected (6.0 × 10^7^ cells), and they were suspended in 200 μl of 0.5 M EDTA (pH 8.0), 800 μl of 1.2 M sorbitol, 10 μl of β-mercaptoethanol, and 20 μl of 50 mg/ml Zymolyase 100T. The cell extract was incubated at 37°C for 45 min, centrifuged at 4500 *g* for 5 min, and the supernatant was removed. The pellet was suspended in 500 μl of extraction solution [50 mM EDTA (pH 8.0), 50 mM Tris–HCl (pH 8.0), 0.5% w/v SDS] and 10 μl of 10 mg/ml proteinase K (Nacalai), incubated at 65°C for 1 h, then 200 μl of 5 M potassium acetate was added and the mixture was incubated on ice for 10 min. The extract was centrifuged for 15 min, the supernatant was transferred, and 500 μl of isopropanol was added. The mixture was centrifuged at 16 000 *g* for 5 min, and the supernatant was discarded. The pellet was washed with 70% ethanol, dried, then resuspended in 300 μl of TE, and 1.5 μl of 20 mg/ml RNase A (Macherey-Nagel) was added and incubated at 37°C for 1 h.

### Yeast protein preparation

Yeast protein was extracted as described previously [20] with slight modification. Briefly, logarithmic phase cells were collected, and the cells were resuspended in 250 μl of water, and mixed with 37.5 μl of alkali solution (92.5% v/v 2 N NaOH, 7.5 % v/v β-mercaptoethanol), then incubated on ice for 10 min. The mixture was then combined with 50% v/v trichloroacetic acid, incubated on ice for 10 min, and centrifuged at 10 000 *g* for 5 min. The supernatant was removed, and the pellet was resuspended in SDS loading buffer [300 mM Tris–HCl (pH 6.8), 600 mM β-mercaptoethanol, 12% w/v SDS, 0.3% w/v Bromophenol blue, 30% v/v glycerol] to 5.0 × 10^6^ cells/μl. The mixture was incubated at 65°C for 5 min. If cycloheximide (CHX) was used, it (water was added for the negative control) was added to the medium at a concentration of 100 μg/ml 2 h before cell collection.

### Western blot analysis

Western blot was performed as described previously [20] with slight modification. The yeast protein sample was centrifuged at 21 130 *g* for 1 min before application. A volume of 5–10 μl of supernatant was applied to 5–20% e-PAGEL (Atto). Electrophoresis was performed in 500 ml of 1× SDS–polyacrylamide gel electrophoresis (PAGE) buffer (25 mM Tris base, 192 mM glycine, and 0.1% w/v SDS) at 10–20 mA. Proteins were transferred to an Immobilon-P polyvinylidene fluoride (PVDF) membrane (Merck Millipore) in 1× transfer buffer (25 mM Tris base, 192 mM glycine, and 10% v/v methanol) at 100 V for 60 min at 4°C. The membrane was soaked in 25 ml of 5% skim milk powder in 1× PBS, 0.05% Tween-20 (PBS-T) for 30 min at room temperature, and then soaked in 10 ml of PBS-T with primary antibody overnight at 4°C. After that, the membrane was washed with 50 ml of PBS-T for 30 min twice. If a secondary antibody was necessary, the washed membrane was soaked in 10 ml of PBS-T with secondary antibody for 1 h at room temperature and washed with 50 ml of PBS-T for 30 min twice. Protein signals were activated with Immobilon Western Chemiluminescent HRP Substrate (Merck Millipore) and photographed. Stripping was performed using 20 ml of Restore™ Western Blot Stripping Buffer (Thermo Fisher Scientific) for 15 min to detect tubulin for normalization. The membrane was washed with 25 ml of PBS-T for 5 min three times. The procedures following the blocking step were performed in the same manner as for the primary antibody. The target protein signals were normalized to tubulin signals in the quantification (target protein/tubulin). In Supplementary Fig. S3A, this value for young CHX– was normalized to 1, and degradation levels were calculated by subtracting the value for CHX+ from that for CHX– in both young and old cells.

Antibodies used for western blotting were as follows.

Sir2: goat polyclonal anti-Sir2 (yN-19) (Santa Cruz Biotechnology)

Goat IgG-HRP: donkey polyclonal anti-goat IgG-HRP (Santa Cruz Biotechnology)

Tubulin: rat monoclonal anti-tubulin alpha:HRP (clone YL1/2) (Bio-Rad)

HA-HRP: mouse monoclonal anti-HA-tag (F-7) HRP (Santa Cruz Biotechnology

These antibodies are also listed in Supplementary Table S3.

### Chromatin immunoprecipitation

Logarithmic phase cells were collected, suspended in 10 ml of PBS, and 278 μl of 37% formaldehyde solution (FUJIFILM Wako) was added. The mixture was incubated for 15 min at room temperature, mixed with 500 μl of 2.5 M glycine, and incubated for 5 min at room temperature. The cells (almost 1–3 × 10^7^ cells) were washed with 500 μl of PBS and lysed with 300 μl of buffer 1 [140 mM NaCl, 0.1% w/v sodium deoxycholate, 50 mM HEPES-KOH (pH 7.5), 1 mM EDTA (pH 8.0), 1% v/v Triton X-100, and Complete EDTA-free Protease Inhibitor Cocktail (Roche)] by zirconia grinding beads (YTZ-ball) with a diameter of 0.5 mm (Nikkato) and a Multi Beads Shocker (Yasui Kikai) for 30 s five times. The lysate was sonicated using a Bioruptor (Cosmo Bio) for 30 s five times at high speed and centrifuged at 9391 *g* for 5 min. The supernatant was mixed with 20 μl of Dynabeads Protein G (Thermo Fisher Scientific), 1 μl of antibody, and buffer 1 (up to a total volume of 321 μl). The mixture was incubated at 4°C overnight, washed with 1 ml of buffer 1 twice, 1 ml of buffer 1′ [500 mM NaCl, 0.1% w/v sodium deoxycholate, 50 mM HEPES-KOH (pH 7.5), 1 mM EDTA (pH 8.0), and 1% v/v Triton X-100] twice, 1 ml of wash buffer [250 mM NaCl, 0.5% w/v sodium deoxycholate, 10 mM Tris–HCl (pH 8.0), 0.5% Nonidet P-40] twice, and 1 ml of 1× TE (pH 8.0) once. The pellet was resuspended in 50 μl of 1× TE (pH 8.0), mixed with 1 μl of 20 mg/ml RNase A (Macherey-Nagel), and incubated at 37°C for 10 min. After that, 50 μl of 1% w/v SDS and 1 mg/ml proteinase K (Nakalai) was added and incubated at 42°C for 30 min, then at 65°C for 3 h. The DNA was extracted by NucleoSpin® Gel and PCR Clean-up kit (Macherey-Nagel).

Antibodies used for ChIP were as follows.

Sir2: goat polyclonal anti-Sir2 (yN-19) (Santa Cruz Biotechnology)

Histone H3: rabbit polyclonal anti-histone H3 (Abcam)

Histone H3K9Ac: rabbit polyclonal anti-acetyl-histone H3 (Lys9) (Millipore)

Histone H3K14Ac: rabbit polyclonal anti-acetyl-histone H3 (Lys14) (Millipore)

Histone H4K16 Ac: rabbit polyclonal anti-histone H4K16 Ac (Active Motif)

RNA polymerase II: rat monoclonal RNA pol II antibody (mAb) (Active Motif)

These antibodies are also listed in Supplementary Table S3.

### Polysome profiling

#### Sucrose density gradient centrifugation

Before cell collection, CHX was added to logarithmic phase cells cultures at a final concentration of 100 μg/ml, incubated for 5 min, and cells were collected. A 300 μl aliquot of sucrose density gradient centrifugation (SDG) lysis buffer [20 mM HEPES-KOH (pH 7.4), 100 mM KOAc, 2 mM Mg(OAc)_2_, 1 mM dithiothreitol (DTT), 1 mM phenylmethylsulfonyl fluoride (PMSF), 100 μg/ml CHX, and cOmplete™ Mini EDTA-free Protease Inhibitor Cocktail (Roche) 1 tablet/10 ml] was added to the cells and they were ground in liquid nitrogen using a mortar. The suspension was centrifuged at 5600 *g* for 10 min at 4°C. The supernatant was transferred to a new tube and centrifuged at 20 080 *g* for 10 min at 4°C. The supernatant was again transferred to a new tube and centrifuged at 20 080 *g* for 20 min at 4°C. The cell lysate was layered onto a 10–50% sucrose gradient prepared in 10 mM Tris-acetate (pH 7.5), 70 mM NH_4_OAc, and 4 mM Mg(OAc)_2_ in 14 × 95 mm polyallomer tubes (SETON SCIENTIFIC) using a Gradient Master (Biocomp Instruments). Samples were centrifuged at 285 000 *g* in an SW40Ti rotor (Beckman Coulter) for 2 h at 4°C and then fractionated using a Piston Gradient Fractionator™ (Biocomp Instruments). Polysome profiles were generated by continuous absorbance measurement at 260 nm.

#### RNA isolation

A 226 μl aliquot from each fraction was collected for RNA isolation. Each fraction was mixed with 500 μl of 8 M guanidine-HCl, and 750 μl of 100% EtOH was added. After mixing, the mixture was incubated at –30°C overnight. The mixture was then centrifuged at 20 080 *g* for 15 min at 4°C and the supernatant was discarded. Next, 300 μl of 75% EtOH was added, the mixture was centrifuged at 20 080 *g* for 15 min at 4°C, and the supernatant was discarded again. Then 200 μl of RNA buffer [20 mM Tris–HCl (pH 7.5), 10 mM EDTA (pH 8.0), 0.3 M NaCl, and 1% SDS)] and 20 μl of 3 M NaOAc were added and mixed. A 600 μl aliquot of 100% EtOH was added, and the mixture was incubated at –30°C for 1 h. The mixture was centrifuged at 20 080 *g* for 15 min at 4°C, and the supernatant was discarded. Next, 300 μl of 70% EtOH was added, and the mixture was centrifuged at 20 080 *g* for 15 min at 4°C. After discarding the supernatant, the pellet was dried and resuspend in 20 μl of diethylpyrocarbonate (DEPC)-treated water.

### Quantitative PCR analysis

**Figure 2.**
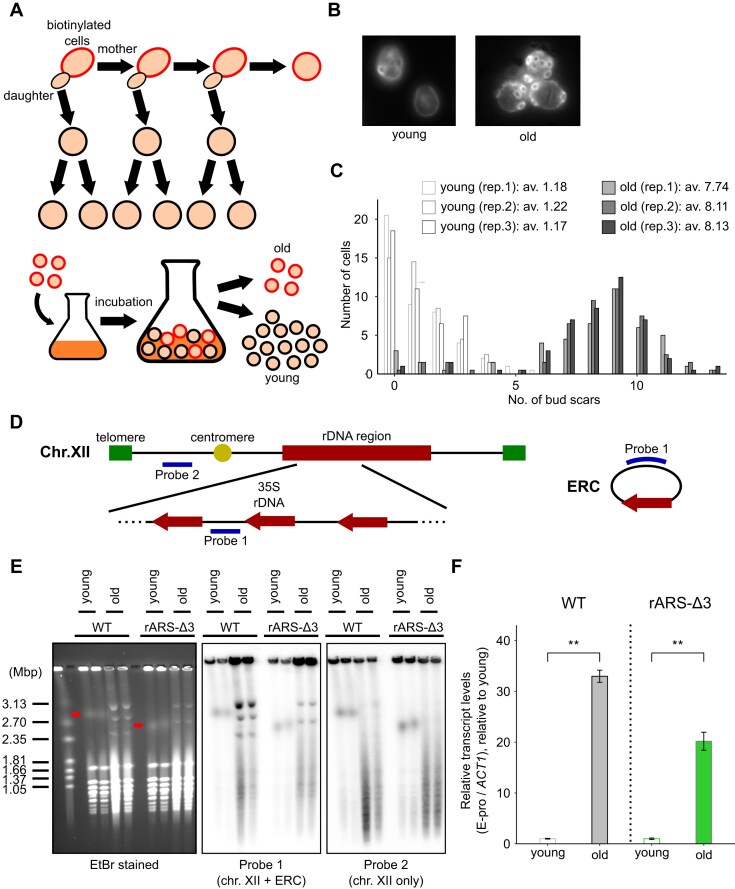
rDNA instability and E-pro activation in old cells. (**A**) Schematic diagram illustrating the sorting of old cells using biotin and streptavidin beads. (**B**) Young and old cells sorted using this method. White circles indicate bud scars stained with Calcofluor white. (**C**) Comparison of the number of bud scars between young and old cells (*n* = 90 for each group). The bar represents the cell count. (**D**) Positions of probes used for Southern blotting. (**E**) PFGE analysis of young and old cells. The gel was stained with EtBr (left), and Southern blotting was performed using probe 1 (middle) and probe 2 (right), as indicated in (D). The size marker is the *H. wingei* chromosome. Red arrowheads suggest chr. XII in young cells. (**F**) Comparison of E-pro transcript levels between young and old cells (*n* = 3). Data for the wild-type strain (left) and the rARS-Δ3 strain (right) are shown. Data are represented as the mean ± SEM. Statistical comparisons were performed using a paired *t*-test (**P < 0.01).

**Figure 3.**
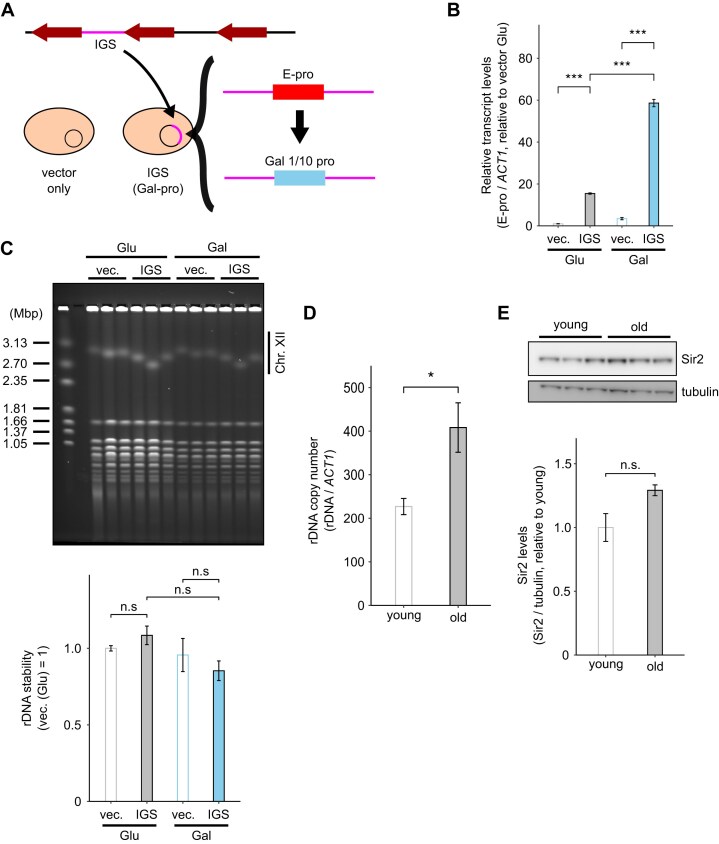
The effect of non-coding RNA (ncRNA) from the IGS region on rDNA instability and the change of Sir2 levels with aging. (**A**) Schematic of the IGS (Gal-pro) strain. The strain was transformed with plasmids that contain the IGS region, in which E-pro was replaced with the Gal 1/10-inducible promoter. (**B**) Transcripts from the Gal-pro plasmid and E-pro in the genome (*n* = 3). Data are represented as the mean ± SEM. Statistical comparisons were performed using Tukey's multiple comparison test (***P < 0.001). (**C**) PFGE analysis of rDNA stability. The gel was stained with EtBr (top). The size marker is the *H. wingei* chromosome. Quantification of rDNA instability was performed using the gel (bottom). Data are represented as the mean ± SEM. Statistical comparisons were performed using Tukey's multiple comparison test (n.s. *P* ≥ 0.05). (**D**) Comparison of rDNA copy number (genome and ERC) in young and old cells (*n* = 3). Data are represented as the mean ± SEM. Statistical comparisons were performed using a paired *t*-test (*P < 0.05). (**E**) Comparison of Sir2 levels in young and old cells by western blotting. Tubulin was used as a loading control. Data are represented as the mean ± SEM. Statistical comparisons were performed using a paired *t*-test (n.s. *P* ≥ 0.05).

**Figure 4.**
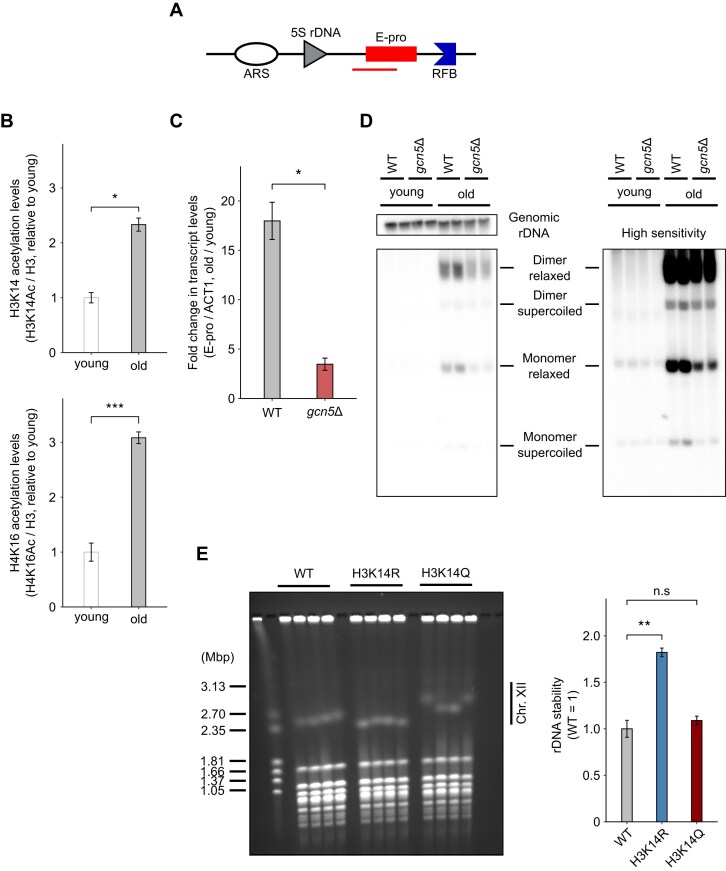
Analyses of histone acetylation and rDNA instability. (**A**) Positions of the regions detected by qPCR primers for ChIP-qPCR analysis, as indicated by the red bar. (**B**) Acetylation levels of histone H3K14 (top) and H4K16 (bottom) in young and old cells determined by ChIP-qPCR (*n* = 3). Data are represented as the mean ± SEM. Statistical comparisons were performed using a paired *t*-test (*P < 0.05, ***P < 0.001). (**C**) Fold change in E-pro transcript levels with aging (*n* = 3). Data are represented as the mean ± SEM. Statistical comparisons were performed using Welch's *t*-test (*P < 0.05). (**D**) ERC levels in young and old cells. Short exposure (left) was used for old cells to avoid saturation, and long exposure (right) was used for young cells. (**E**) PFGE analysis of rDNA stability (left). The gel was stained with EtBr. The size marker is the *H. wingei* chromosome. Quantification of rDNA instability was performed using the gel (right). Data are represented as the mean ± SEM. Statistical comparisons were performed using Dunnett's *t*-test (n.s. *P* ≥ 0.05, **P < 0.01).

**Figure 5.**
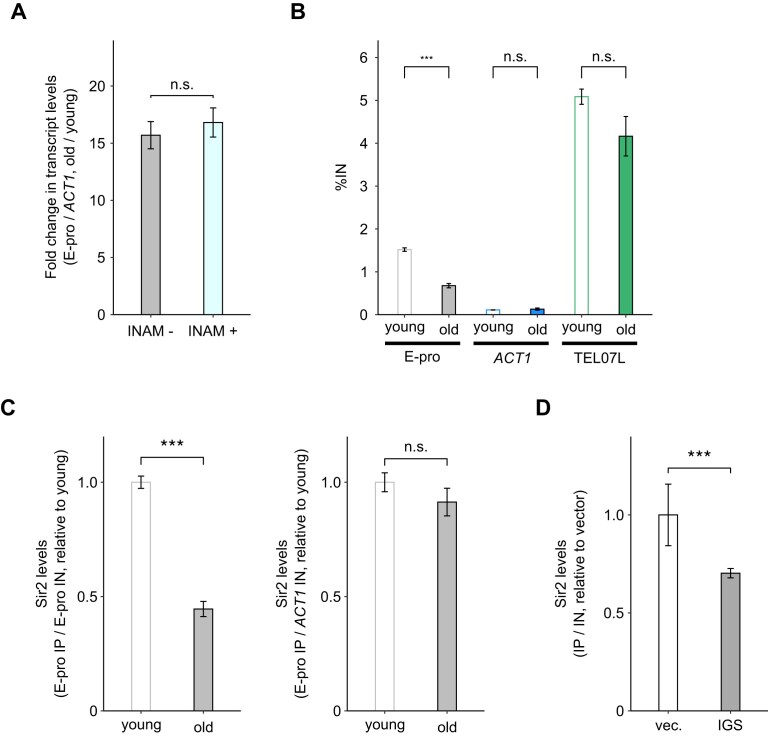
Analyses of changes in Sir2 activity and localization with aging. (**A**) Fold change in E-pro transcript levels with aging with or without INAM treatment (*n* = 3). Data are represented as the mean ± SEM. Statistical comparisons were performed using Welch's *t-*test (n.s. *P* ≥ 0.05). (**B**) Sir2 levels in young and old cells (*n* = 3). The *ACT1* region and the TEL07L region served as negative and positive controls for the quantification, respectively. Data are represented as the mean ± SEM. Statistical comparisons were performed using a paired *t*-test (n.s. *P* ≥ 0.05, ***P < 0.001). (**C**) Sir2 levels in young and old cells (*n* = 3), derived from the same dataset as in (B). Immunoprecipitated DNA (IP) for E-pro was normalized to input DNA (IN) for E-pro (left) or *ACT1* (right). Data are represented as the mean ± SEM. Statistical comparisons were performed using a paired *t*-test (n.s. *P* ≥ 0.05, ***P < 0.001). (**D**) Sir2 levels at E-pro in the strain shown in Fig. 3A cultured in glucose-containing medium (*n* = 3). Data are represented as the mean ± SEM. Statistical comparisons were performed using the permutation Brunner–Munzel test (***P < 0.001).

qPCR was performed using a Thermal Cycler Dice® Real Time System II (TaKaRa) or a QuantStudio 1 Real-Time PCR System (Thermo Fisher Scientific) with THUNDERBIRD SYBR qPCR Mix (TOYOBO) or THUNDERBIRD Next SYBR qPCR Mix (TOYOBO) under the following conditions: denaturation at 95°C for 30 s, followed by 40 cycles of 95°C for 5 s and 60°C for 30 s. Primer sequences used for qPCR were as follows :

E-pro transcription (Figs 2F, 3B, 4C, and 5A; Supplementary Fig. S4C) (5′-CCCATAACTAACCTACCATTCGA and 5′-TCAAGTAGTAGCAACCCAATGAG)

*ACT1* mRNA and *ACT1* region (Figs 2F, 3B, D, 4C, and 5A–C; Supplementary Figs S3B, D, S4A, C, and S5D) (5′-CGAATTGAGAGTTGCCCCAG and 5′-CAAGGACAAAACGGCTTGGA)

E-pro region (Figs 3D, 4B, and 5B–D; Supplementary Figs S4A and S5C) (5′-GCGGTATGCGGAGTTGTAAG and 5′-CGGTTTTGTTCTCTTCCCTCC)

*SIR2* mRNA (Supplementary Fig. S3B, D) (5′-CCGAGGATTTGAACTCGTTATAC and 5′-CCAAATCTTGAACACGCTCTTGC)

*GCN5* mRNA (Supplementary Fig. S5D) (5′-CATCAGATTGAAGAGGATCACTTG and 5′-GGTGCCCTCTTGTTTATTGGTCTC)

TEL07L (Fig. 5B) (5′-AACCACCATCCATCTCTCTACTTACTACTA and 5′-AGAACAACAGTACAGTGAGTAGGACATG).

These primers are also listed in Supplementary Table S4. For quantification except Fig. 5B, we calculated ΔCt (target Ct – standard Ct) and determined relative expression using the 2^(–ΔCt)^ method. The average of these values in the control group was normalized to 1. In Fig. 5B, after calculating 2^(–ΔCt)^, the percentage of input DNA (%IN) was derived.

## Quantification and statistical analysis

Statistical analyses were performed using R (ver. 4.4.1). The specific statistical analyses performed are indicated in the figure legends. *n* represents the number of cells in Fig. 2C and the number of colonies in the other figures. The bars in the graphs represent mean values, and the error bars indicate the standard error of the mean (SEM), in experiments performed in multiple replicates unless otherwise noted in the figure legends.

For band quantification, Fiji [46] (ver. 2.16.0) was used with the Band/Peak Quantification Tool (Kenji Ohgane, Hiromasa Yoshioka 2019) [47]. Quantification of gel bands was carried out by an ImageJ Macro, Band/Peak Quantification Tool. protocols.io, https://dx.doi.org/10.17504/protocols.io.7vghn3w).

## Results

### Changes in rDNA instability and E-pro activation with aging

To investigate rDNA instability in old cells, we harvested them using a biotin–streptavidin strategy [41] (Fig. 2A). Briefly, yeast cell surfaces are labeled with biotin, which remains only on mother cells after budding. Thus, labeled old cells were harvested by capturing with streptavidin after cultivation, while the remaining cells were used as young cells. Using this strategy, old cells were accurately separated (∼7–8 divisions) (Fig. 2B, C), and subsequently used for analysis.

First, we compared rDNA instability between young and old cells using PFGE to detect rDNA copy number fluctuations. Southern blotting was performed to identify chr. XII, employing two probes. Probe 1 (rDNA probe) detects the rDNA sequence, identifying both chr. XII and ERCs, whereas Probe 2 (non-rDNA probe) detects the short arm region of chr. XII (Fig. 2D), identifying chr. XII only.

We also utilized the rARS-Δ3 strain, in which a non-essential element of the replication origin (ACS-III) is deleted, significantly reducing replication initiation efficiency, particularly in ERCs [40]. As a result, replication of ERCs is rare, and their numbers are considerably lower than in the wild-type strain. In the rARS-Δ3 strain, the status of genomic rDNA is more clearly observed, as the effect of ERCs is minimized. In the Southern blotting experiment (Fig. 2E), sharp signals were detected only in old cells when using Probe 1 as well as the EtBr-stained gel. These signals were absent with Probe 2 and were weaker in the rARS-Δ3 strain compared with the wild-type strain, suggesting that these signals represent ERCs. Furthermore, to confirm this, exonuclease V treatment was performed before PFGE to digest linear DNA (Supplementary Fig. S1). The sharp signals persisted while other chromosomal signals almost disappeared, suggesting that they consist of circular DNA. This result further supports the idea that these signals represent ERCs. However, the apparent size of these circular DNAs was comparable with that of chromosomes, leading us to consider that their exact size could not be accurately measured under the current PFGE conditions. This behavior is consistent with previous studies on PFGE, which demonstrated that the mobility of supercoiled circular DNA is largely independent of pulse time, causing it to migrate differently from large linear chromosomes [48, 49]. Finally, referring to Southern blotting analysis, rDNA instability was observed in old cells in both strains, as indicated by Probe 2. These results are consistent with those of previous studies [50, 51].

Since previous research has shown that rDNA instability is caused by the activation of E-pro transcription [16, 17], we measured E-pro transcript levels using quantitative reverse transcription–PCR (RT–qPCR). The results showed that E-pro transcript levels increased with aging. In old cells, E-pro transcripts were ∼30-fold higher in the wild-type strain and 20-fold higher in the rARS-Δ3 strain compared with young cells (Fig. 2F), consistent with earlier findings [50]. These results indicate that in old cells, E-pro on the chromosome is activated, leading to increased rDNA instability.

### The effect of increased transcripts from the IGS region and the change of Sir2 levels with aging

As shown above, E-pro transcript levels increased with aging, which is suggested to contribute to rDNA instability. However, it remains unclear whether the transcripts themselves or the transcriptional process cause rDNA instability. To address this question, we introduced plasmids containing the IGS region of rDNA, where the E-pro was replaced by the Gal 1/10 promoter (Gal-pro), into yeast (Fig. 3A), while the genomic E-pro remains unchanged. Gal-pro transcript levels from the IGS region were controlled by the sugar in the medium. We used glucose medium as a negative control alongside galactose medium. Additionally, a strain introduced with only vector plasmids was used as another negative control. These two strains were incubated for ∼30 generations in the presence of the appropriate sugar after their construction to observe changes in rDNA before being used for experiments.

RT–qPCR results showed that Gal-pro transcript levels increased in the galactose medium (Fig. 3B). These strains were then analyzed using PFGE. No differences in the sharpness of chr. XII signals were observed. Moreover, the signal intensity per unit area was calculated for quantification (a simplified explanation is provided here), but no significant difference was detected (Fig. 3C). Details of the method are provided in the Materials and methods. Previous studies showed that transcription activation by replacing E-pro with Gal-pro in the genome causes rDNA instability [16, 17]. However, our experiment showed that the increase in transcript levels from the plasmids does not affect rDNA stability. These results suggest that either E-pro transcripts act solely in *cis*, or that rDNA instability is caused by the act of E-pro transcription itself rather than by the resulting transcripts, a perspective that aligns with the model proposed in the previous study [17].

We then investigated the cause of the increase in E-pro transcript levels. It is known that the number of ERCs, which include the E-pro region, is higher in old cells than in young cells [36], raising the possibility that the increase in E-pro transcript levels may be caused by an increase in ERCs. To test this, we determined the rDNA copy number (genomic rDNA + ERC) by qPCR. This analysis revealed that rDNA copy number in old cells was approximately twice as high as in young cells (Fig. 3D), a ratio much lower than that of E-pro transcript levels (>30-fold) (Fig. 2F, left). Furthermore, E-pro transcript levels increased with aging in the rARS-Δ3 strain, which has lower ERC levels than the wild-type strain (Fig. 2F, right). The findings suggest that the increase in E-pro transcript levels is caused by transcriptional activation, not ERC accumulation.

To explore the cause of E-pro transcription activation, we focused on Sir2. Previous studies reported that Sir2 maintains rDNA stability by regulating E-pro transcription [17, 18] and decreases with aging [32]. In contract, our experiment showed that Sir2 levels did not decrease with aging (Fig. 3E; Supplementary Fig. S2A–D), as determined by western blotting. Furthermore, Sir2 protein stability (Supplementary Fig. S3A), *SIR2* mRNA levels (Supplementary Fig. S3B), and *SIR2* translation efficiency (Supplementary Fig. S3C, D) were analyzed using western blotting after treatment with CHX, which inhibits protein synthesis, RT–qPCR, and polysome profiling followed by RT–qPCR, respectively. None of these analyses provided data supporting a decrease in Sir2 levels with aging. In fact, *SIR2* mRNA levels were slightly increased with aging (Supplementary Fig. S3B). These findings align with the previous report [20] suggesting that increased rDNA copies enhance *SIR2* transcription. This occurs because UAF, which represses *SIR2* expression, is titrated by the rDNA copies (see Introduction). Furthermore, Rrn5—the least abundant protein in UAF [52]—shows decreased levels with aging (Supplementary Fig. S3E). Moreover, to further confirm our results, we normalized Sir2 protein levels to the nucleolar protein Rrn5 and found that the Sir2/Rrn5 ratio did not decrease with aging (Supplementary Fig. S3E).

### The relationship between histone acetylation in the E-pro region and rDNA instability

Under our experimental conditions, Sir2 levels were not affected by aging, suggesting that factors other than Sir2 contribute to E-pro transcription activation. Based on previous studies [27, 28], we examined histone modifications by chromatin immunoprecipitation (ChIP)-qPCR. The results showed that histone H3K14 and H4K16 acetylation levels increased with aging (Fig. 4A, B). Furthermore, we performed ChIP-qPCR to analyze RNA polymerase II (Pol2) localization in E-pro. We used input DNA of *ACT1* for normalization to remove the effect of increased rDNA copy number with aging. We revealed that the amount of Pol2 which combines with E-pro increases with aging (Supplementary Fig. S4A).

Histone H4K16 acetylation is known to affect E-pro transcription and rDNA instability [31]. In contrast, the role of H3K14 acetylation has not been reported previously. In budding yeast, Sas3 and Gcn5 function as histone H3K14 acetylases [53–55]. To investigate the effects of histone H3K14 acetylation on rDNA stability, we established *sas3* and *gcn5* deletion mutants (*sas3*Δ and *gcn5*Δ, respectively). PFGE analysis showed no significant differences in rDNA stability in either young or old *sas3*Δ or *gcn5*Δ cells (Supplementary Fig. S4B). In *sas3*Δ, no significant changes in the fold change of E-pro transcript levels were observed. Thus, further analyses of *sas3*Δ were not performed (Supplementary Fig. S4C).

In contrast, the fold change in E-pro transcript levels with aging was ∼3.5-fold in the *gcn5*Δ strain compared with 18-fold in the wild-type strain (Fig. 4C). We speculated that rDNA in old cells might be too unstable to detect the difference using PFGE. To address this, we performed an ERC assay. ERC levels, which reflect rDNA instability, were lower in the *gcn5*Δ strain compared with the wild-type strain in old cells but not in young cells (Fig. 4D; Supplementary Fig. S5B).

ERC levels are influenced not only by rDNA stability but also by the replicative initiation activity, as observed in the rARS-Δ3 strain. Interestingly, ERC levels in young *gcn5*Δ cells were not significantly reduced compared with wild-type cells, a phenotype that differs from the rARS-Δ3 strain, in which ERC levels are reduced in young cells (Supplementary Fig. S5A, B), as reported in a previous study [51]. These findings suggest that rDNA in the *gcn5*Δ strain is more stable than in the wild-type strain in old cells.

Given that Gcn5 acetylates not only H3K14 but also H3K9 [53, 56], we proceeded to investigate H3K9 acetylation. While H3K9 acetylation levels showed an increasing trend, they did not reach statistical significance (Supplementary Fig. S5C).

Additionally, H3K14R and H3K14Q mutants, which are non-acetylatable and mimic acetylation, respectively, were analyzed using PFGE. The chr. XII signals in the H3K14R strain were sharper than those in the wild-type strain. Moreover, quantification of signal intensity per unit area (simplified explanation provided here) indicated that rDNA in the H3K14R strain was more stable than in the wild-type strain (Fig. 4E). Details of the method are provided in the Materials and methods. In contrast, no difference in rDNA instability was observed between the H3K14Q strain and the wild-type strain.

*GCN5* expression levels did not change with aging (Supplementary Fig. S5D, E). While rDNA stability improved in the *gcn5*Δ mutant, it remained unstable in old cells compared with young cells. This suggests the involvement of additional factors that influence rDNA instability in old cells.

### The changes in Sir2 activity and localization with aging

In summary, Sir2 protein levels do not decrease with aging, but the acetylation levels of H3K14 and H4K16 in the E-pro region increase. Since these histone residues are deacetylated by Sir2, we next examined the enzymatic activity of Sir2. First, INAM treatment was performed to enhance Sir2 activity by increasing NAD^+^ levels and counteracting the inhibitory effect of NAM on Sir2 activation [34, 35]. However, this treatment did not affect the fold change in E-pro transcript levels with aging (Fig. 5A). These results suggest that Sir2 activity is sufficient for deacetylation in both young and old cells.

Next, we analyzed Sir2 localization using ChIP-qPCR. Sir2 association with E-pro in old cells was approximately half that in young cells when normalized to the input DNA of E-pro. However, no significant difference was observed when normalized to the input DNA of the *ACT1* region (Fig. 5B, C). We interpreted these results as being caused by an increase in rDNA copy number with aging (Fig. 3D). Specifically, the amount of Sir2 on a rDNA copy does not change with aging. However, as the number of rDNA copies increases due to ERC accumulation, the relative amount of Sir2 bound to genomic rDNA decreases, leading to histone acetylation.

Supporting this hypothesis, plasmids containing the IGS region but modified with a galactose-inducible promoter (Gal-pro) instead of E-pro increased transcript levels from the IGS region, even though the Gal-pro in these plasmids was inactive in glucose (Fig. 3B), suggesting that E-pro transcription in genomic rDNA is activated. As the Gal-pro plasmid includes the RFB which is associated with Sir2 [30], Sir2 may be sequestered. In line with this, the localization levels of Sir2 at E-pro decreased when the IGS region was transformed by these plasmids (Fig. 5D). Moreover, in the rARS-Δ3 strain, which exhibits lower ERC proliferation than the wild-type strain, the fold change in E-pro transcript levels with aging was lower than in the wild-type strain (Fig. 2F). The reason for this may be that the lower rDNA copy number, resulting from reduced ERC proliferation, affects not only the amount of template DNA but also the extent to which Sir2 decreases on genomic rDNA. Taken together, these findings suggest that Sir2 activity does not decrease, at least by the midpoint of the yeast lifespan when rDNA instability occurs. Instead, its activity within genomic rDNA is reduced due to ERCs sequestering Sir2.

## Discussion

**Figure 6.**
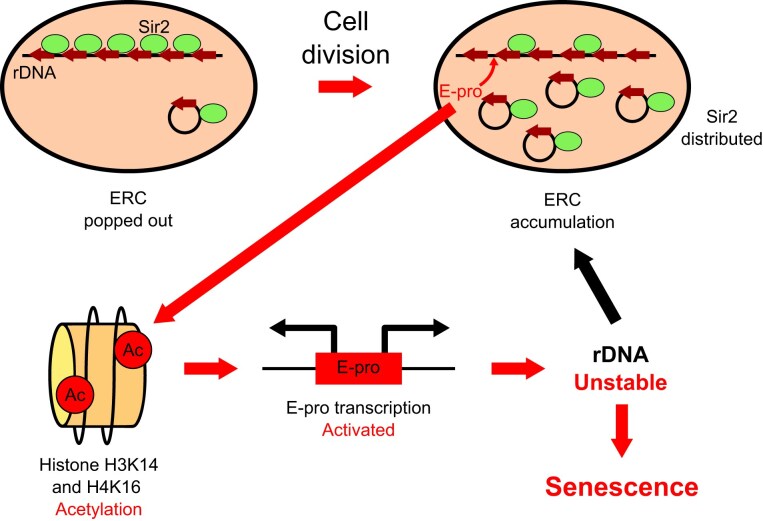
The model of cellular senescence via rDNA instability. See the text for details.

Our findings suggest the mechanism of cellular senescence. In old cells, Sir2 on genomic rDNA is depleted by accumulated ERCs, preventing the deacetylation of histones H3K14 and H4K16. This leads to the activation of E-pro transcription, rDNA destabilization, and the induction of cellular senescence (Fig. 6). ERCs increase rapidly because they contain replication origins (ARSs) and remain in the mother cell after cell division, as they lack a centromere that ensures equal segregation to daughter cells. Once an ERC is generated from rDNA, it replicates and accumulates in the mother cell, triggering senescence. Furthermore, previous reports have shown that Sir2 overexpression extends the lifespan and enhances rDNA stability [19, 40]. Therefore, we hypothesized that Sir2 depletion would severely compromise longevity and rDNA stability.

Although our model posits that histone acetylation activates transcription at E-pro, the increase in Pol2 levels at E-pro was only 2-fold. This is notably lower than the observed increase in E-pro transcript levels (Supplementary Fig. S4A; Fig. 2F). This discrepancy suggests the involvement of other factors contributing to the elevated transcript levels. These may include, for example, transcriptional elongation modulated by histone modification or reduced RNA degradation by Trf4, as reported in previous studies [57–59]. Additionally, the age-associated increase in E-pro transcript levels exhibits strain-dependent variation. Specifically, the fold change was ∼30-fold in the NOY408-1b wild-type strain (Fig. 2F), whereas it was <20-fold in the BY4741 wild-type strain (Fig. 4C; Supplementary Fig. S4C).

We analyzed a strain containing IGS region plasmids (Gal-pro). However, we encountered several issues with this strain. Initially, non-coding RNA (ncRNA) transcript levels were ∼4-fold higher in galactose than in glucose, representing a limited induction (Fig. 3B). We hypothesize that two distinct factors contribute to this limited induction. First, there is some ncRNA transcription even in glucose, leading to basal transcription in the absence of full induction. Second, the IGS region on these plasmids may sequester Sir2 from genomic rDNA (Fig. 5D), leading to elevated ncRNA transcription. Consequently, while a certain degree of induction was observed, the overall rate of increase was suppressed. Furthermore, even in the vector-only control strain, ncRNA transcript levels showed variability depending on the sugar source. We were unable to determine the reason for this, indicating that further investigation is needed.

Regarding rDNA, plasmids containing the IGS region (Gal-pro) did not induce rDNA instability (Fig. 3B, C). Based on the mechanism of cellular senescence caused by Sir2 titration, these plasmids should destabilize rDNA because they can deplete Sir2 from genomic rDNA. One possible explanation is that there is a threshold required to induce rDNA instability. The increase in E-pro transcript levels in old cells was >30-fold compared with young cells, whereas the increase caused by plasmids containing the IGS region was <20-fold compared with plasmids without the region in NOY408-1b, which might not be sufficient to destabilize rDNA. Additionally, the copy number of these plasmids (YEps) is 40–80 per haploid genome [60], which is less than the increase in rDNA copy number in old cells (Fig. 3D).

The previous study reported that short rDNA causes suppression of *SIR2* mRNA levels (negative feedback by Sir2 itself) and affects silencing of HM loci and telomeres due to surplus Sir2 [61]. Consistent with this, in our study, the accumulation of ERCs with aging led to excessive rDNA copies, which were associated with an increase in *SIR2* mRNA levels (Supplementary Fig. S3B). Sir2 localization levels at TEL07L showed a decreasing trend with aging; however, this decreasing trend was not statistically significant (Fig. 5B). One possible explanation is that the composition of the Sir2-containing complex differs between rDNA, telomeres, and HM loci. At the rDNA locus, Sir2 forms the RENT complex with Cdc14 and Net1, whereas at telomeres and HM loci, it is part of the SIR complex along with Sir3 and Sir4 [62]. Therefore, if the SIR complex is more stable than the RENT complex, the titration effect by ERCs may be less pronounced at telomeres and HM loci. Another possibility is that approximately eighth generation cells were used as old cells in our experiments. If older cells with higher ERC levels are used, Sir2 depletion may become more apparent in non-rDNA regions. Furthermore, a previous study suggests that silencing of HM loci collapses with aging [63]. Our model may also relate to this observation.

In addition to Sir2, we found that Gcn5 affects rDNA instability in old cells but not in young cells. This effect is likely to be mediated by the histone H3K14 acetylation activity of Gcn5, as the fold change in E-pro transcript levels with aging in the *gcn5*Δ strain was lower than in the wild-type strain (Fig. 4C). However, acetylation levels at H3K9, which Gcn5 is known to acetylate [53, 56], showed an increasing trend with aging, but it was not statistically significant. We hypothesize that this observation is due to Gcn5's weaker acetylation ability at H3K9 compared with H3K14, as indicated by the previous study [53]. In the ERC assay, ERC levels of the *gcn5*Δ strain did not differ from the wild-type strain in young cells (Fig. 4D). This result diverged from that of the previous study [64]. We considered that this discrepancy could be attributed to factors such as whether old cells were sorted and the uniformity of genomic rDNA copy number.

*GCN5* expression levels did not change with aging (Supplementary Fig. S5D, E). Additionally, *sas3* deletion did not affect rDNA instability or E-pro transcription, despite Sas3 also having H3K14 acetylation activity [55]. Sas3 is a component of the NuA3 complex [65], whereas Gcn5 is a component of two independent histone acetyltransferase (HAT) complexes, the ADA complex and the SAGA complex [66]. It is possible that components of these complexes are also involved in cellular senescence through Gcn5. Additional investigations into the relationship between Gcn5 and E-pro are needed. Furthermore, despite the increased rDNA stability in old *gcn5*Δ cells compared with the wild type, their lifespan was not extended in previous reports [64, 67, 68]. This paradox is attributed to Gcn5's modulation of the retrograde response, which is known to expand lifespan [64].

The previous studies identified ERCs as a cause of cellular senescence because their accumulation provokes GLM and the SEP [36–38]. These phenomena occur near the end of the life course, ∼5 generations before cell death [37, 39]. We identified a new pathway of ERC-induced cellular senescence via rDNA instability, which occurs earlier, at 7–8 generations, compared with the previously identified pathway. The previous studies suggest that cellular senescence is induced by rDNA instability rather than directly by ERCs [16, 40]. The pathway proposed in this study suggests that ERC accumulation leads to rDNA instability, which in turn induces cellular senescence. Thus, our findings are consistent with previous studies. However, how rDNA instability induces cellular senescence remains unclear. This is an open question that requires further investigation.

Contrary to prior studies [32, 69, 70], Sir2 levels did not decrease with aging in this study. Furthermore, none of the events related to *SIR2* expression suggested a reduction in Sir2 levels (Supplementary Fig. S3). This discrepancy might be due to differences in cell lines or variability in expression levels. For example, in SC medium, some colonies exhibited decreased Sir2 levels with aging (Supplementary Fig. S2C). Thus, specific conditions may affect Sir2 expression. However, we could not identify these conditions and instead focused on investigating other factors that activate E-pro transcription with aging under our experimental conditions.

To investigate the effect of histone modification in the E-pro region, we used gene deletion mutants and histone mutants. However, these mutants affect regions outside E-pro and may function through pathways other than the one proposed in this study (Fig. 6). Additionally, histone mutants may not accurately mimic the deacetylated or acetylated states. For example, rDNA in the H3K14Q strain was as stable as in the wild-type strain (Fig. 4E), contrary to expectations. We suspect that the H3K14Q strain does not accurately mimic acetylation. As the previous study suggested, H3K14 acetylation restricts H3K4 demethylation [71], but the H3K14Q strain does not exhibit this phenomenon [72].

ERCs have not been reported in human cells, but circular extrachromosomal DNA is known to contribute to cancer evolution [73]. Our findings may extend to model organisms beyond yeast, and contribute to uncovering universal mechanisms of aging. Investigating whether DNA structures analogous to ERCs exist in other organisms is a compelling direction for future research.

## Supplementary Material

gkaf923_Supplemental_File

## Data Availability

Original data reported in this paper will be shared at: https://data.mendeley.com/preview/3jj7gf3ttf?a=8c9ac88c-cfb6-4350-ba4d-cd300ff6ef33.

## Supplementary data

Supplementary Data is available at NAR online.
