# Supplementary material for: ERC accumulation depletes Sir2 from rDNA and induces cellular senescence by rDNA destabilization
Source: Nucleic Acids Res. 2025 Oct 29;53(20):gkaf923. doi: 10.1093/nar/gkaf923 (PMC12571442; doi:10.1093/nar/gkaf923)
Supplement: gkaf923_Supplemental_File [file gkaf923_supplemental_file.pdf]

Figure S1

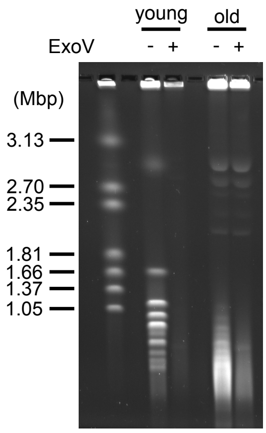

Figure S1: Pulsed field gel electrophoresis (PFGE) analysis of young and old cells following exonuclease V (New England Biolabs) treatment. After preparing agarose plugs, the plugs were washed four times with water for 15 minutes each. Half of the plugs were then incubated in an equilibration mixture (10 µL NEBuffer™ 4, 4 µL 25 mM ATP, and 26 µL water) at 37 °C for 1 hour. Following equilibration, 20 µL (200 U) of exonuclease V was added while negative control plugs received 20 µL of water. All plugs were subsequently incubated overnight at 37 °C. The gel was stained with ethidium bromide (EtBr), and the size marker is *H. wingei* chromosome.

Figure S2

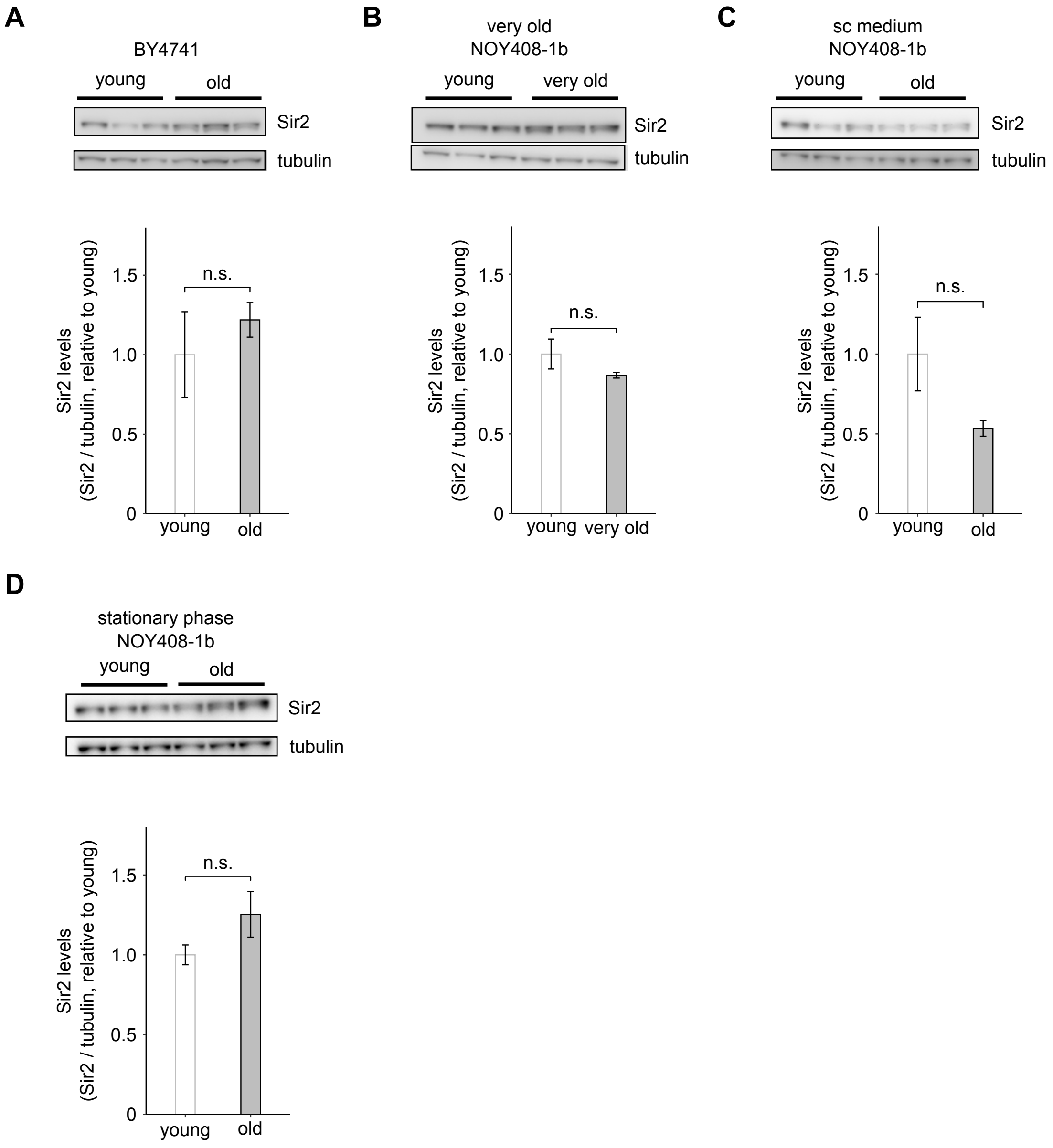

Figure S2: Changes in Sir2 levels with aging under different conditions. (A-D) Western blotting analysis comparing Sir2 levels in young and old cells. The lower panels display the quantification of Sir2 levels. Old cells, defined as those undergoing 7 to 8 divisions, were harvested during the logarithmic phase in YPDA medium (A and B). (A) Sir2 levels in the BY4741 strain. (B) Sir2 levels in very old cells with an average of ~12 divisions in the NOY408-1b strain. (C) Sir2 levels in cells incubated in SC medium in the

NOY408-1b strain. (D) Sir2 levels in cells harvested during the stationary phase in the NOY408-1b strain. Data are represented as mean ± SEM. Statistical comparisons were performed using a paired t-test (n.s.: $p \geq 0.05$).

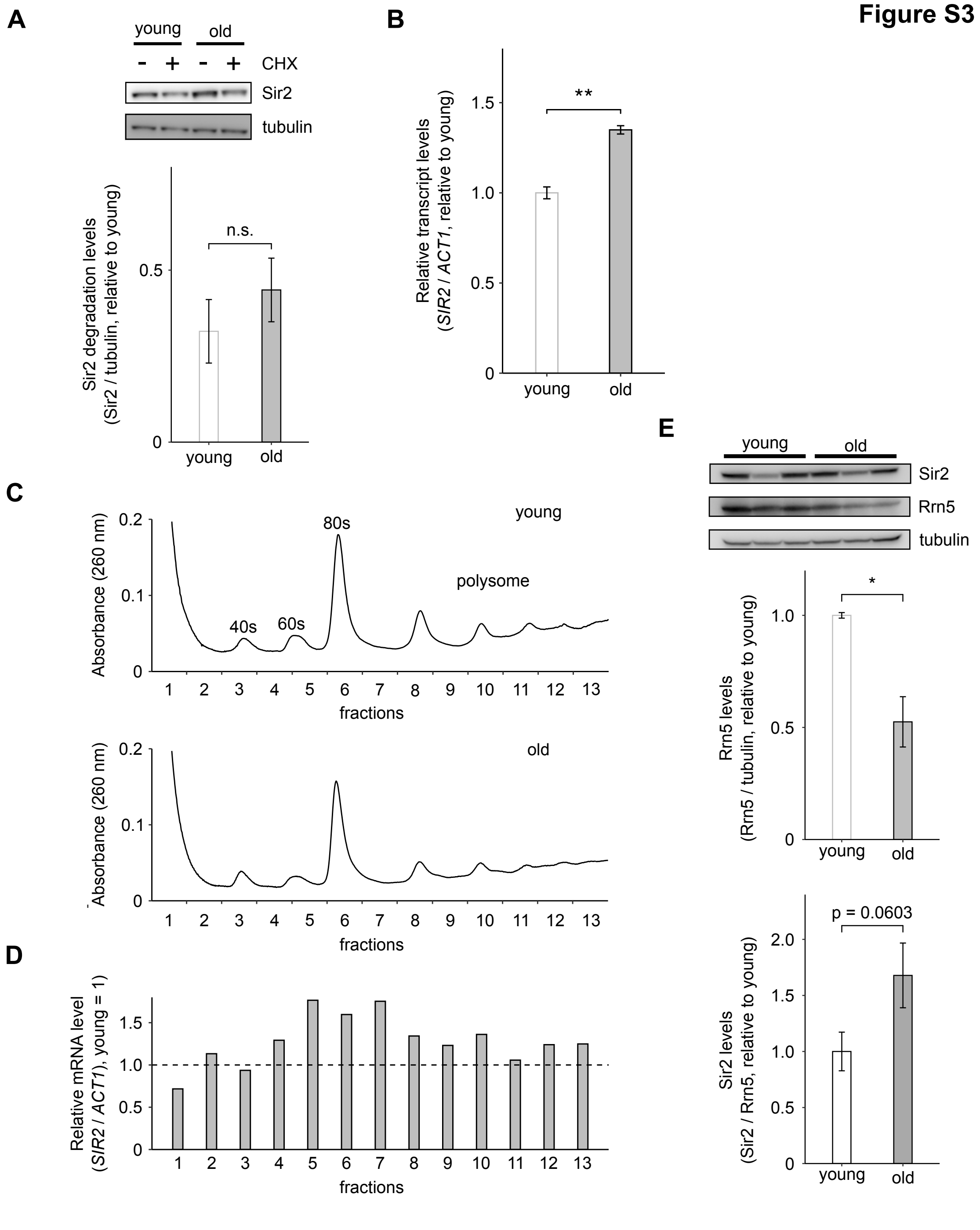

Figure S3: Changes in factors related to Sir2 levels with aging. (A) Sir2 degradation levels in young and old cells. Cycloheximide (CHX), which inhibits protein synthesis, or Milli-Q water was added to the medium 2 hours prior to cell harvesting. For quantification, Sir2 levels in young cells treated with Milli-Q water were normalized to 1. Sir2 degradation levels were calculated by subtracting Sir2 levels under the CHX+ condition from those under the CHX- condition in both young and old cells,

respectively (n = 3). Data are represented as mean ± SEM. The groups were compared using a paired t-test (n.s.: $p \geq 0.05$). (B) *SIR2* mRNA levels in young and old cells (n = 3). Data are represented as mean ± SEM. Statistical comparisons were performed using a paired t-test (**: $p < 0.01$). (C) Polysome profiling of young (top) and old (bottom) cells (n = 1). (D) *SIR2* mRNA levels in the fractions shown in (C) (n = 1). Data are represented as mean. The dotted line represents the levels in young cells (young = 1). (E) Western blot analysis of Rrn5 and Sir2 levels in young and old cells. Rrn5 levels were normalized to Tubulin, while Sir2 levels were normalized to Rrn5. Data are represented as mean ± SEM. Statistical comparisons were performed using a paired t-test (*: $p < 0.05$).

Figure S4

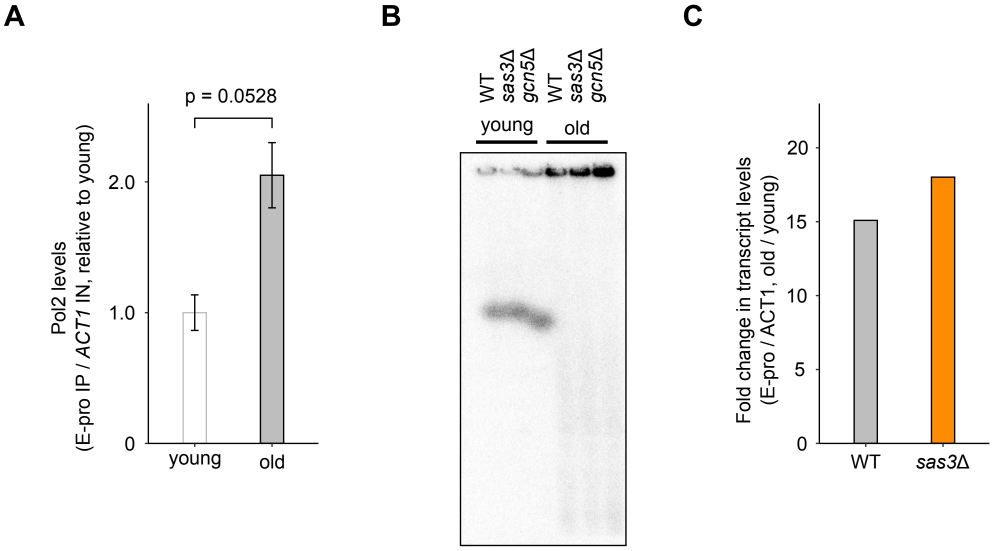

Figure S4: Changes in factors related to transcription. (A) RNA polymerase II levels in young and old cells (n = 3). Immunoprecipitated DNA (IP) for the E-pro was normalized to input DNA (IN) for the ACT1 (right). Data are represented as mean ± SEM. Statistical comparisons were performed using a paired t-test. (B) Southern blot analysis following pulsed-field gel electrophoresis (PFGE) using Probe 2 (see Fig. 2D). (C) Fold change in E-pro transcript levels with aging (n=1).

Figure S5

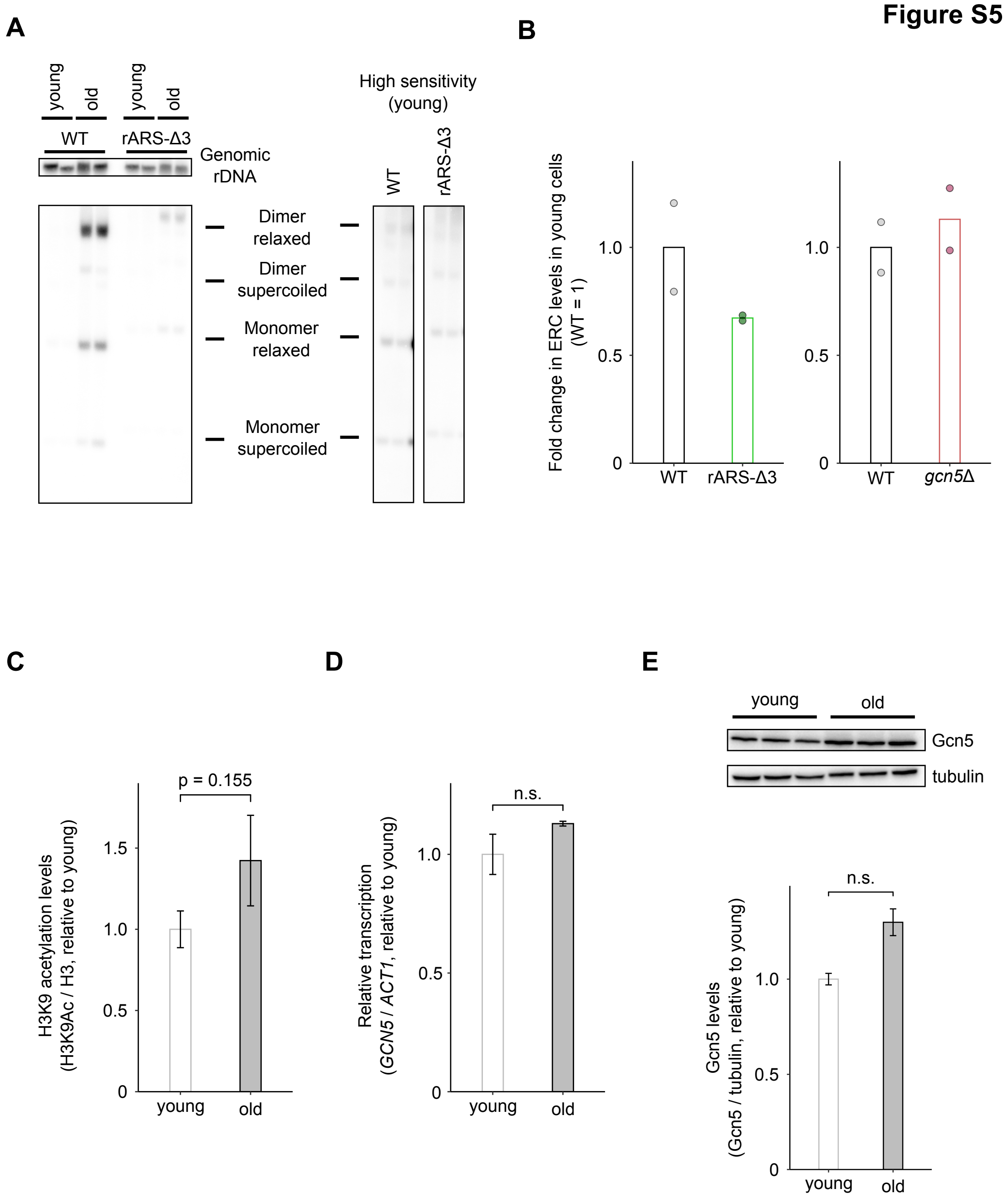

Figure S5: rDNA instability in *gcn5*Δ. (A) Extrachromosomal rDNA circle (ERC) levels in young and old cells. Short exposure (left) was used for old cells to avoid saturation, and long exposure (right) was used for young cells. (B) Fold change in ERC levels in the rARS-Δ3 strain (left) and the *gcn5*Δ strain (right) relative to the wild-type (WT) strain. Quantification was based on data from Fig. S5A (rARS-Δ3) and Fig. 4C (*gcn5*Δ). Data are presented as mean values, with individual dots representing data points. (C) Acetylation

levels of histone H3K9 in young and old cells determined by ChIP-qPCR (n = 3). Data are represented as mean ± SEM. Statistical comparisons were performed using a paired t-test. (D) Fold change in *GCN5* mRNA levels (n = 3). Data are presented as mean ± SEM. (E) Western blot analysis of Gcn5 protein levels in young and old cells. Data are presented as mean ± SEM. Statistical comparisons in (D) and (E) were performed using a paired t-test (n.s.: $p \geq 0.05$).

Table S1: Yeast strains

**genotype**	**source**
S. cerevisiae (strain NOY408-1b *MATa ade2-1, ura3-1, his3-11, 15, trp1-1, leu2-3, 112, can1-100*)	Nogi *et al.* (S1)
S. cerevisiae (strain NOY408-1b ACSⅢ is replaced to *URA3*)	Ganley *et al.* (40)
S. cerevisiae (strain BY4741 *MATa his3Δ1 ura3Δ0 leu2Δ0 met15Δ0*)	ATCC
S. cerevisiae (strain BY4741 *GCN5::kanMX*)	This manuscript
S. cerevisiae (strain BY4741 Gcn5-3HA)	This manuscript
S. cerevisiae (strain NOY408-1b Yeplac181)	This manuscript
S. cerevisiae (strain NOY408-1b Yeplac181-IGS)	This manuscript
S. cerevisiae (strain NOY408-1b Rrn5-3HA)	This manuscript
S. cerevisiae (*MATa his3Δ200 leu2Δ0 lys2Δ0 trp1Δ63 ura3Δ0 met15Δ0 hht1-hhf1::NatMX4 can1::MFA1pr-HIS3 hht2-hhf2::URA3-HHTS-HHFS*)	Horizon Discovery
S. cerevisiae (*MATa his3Δ200 leu2Δ0 lys2Δ0 trp1Δ63 ura3Δ0 met15Δ0 hht1-hhf1::NatMX4 can1::MFA1pr-HIS3 hht2-hhf2::URA3-hhts(K14R)-HHFS*)	Horizon Discovery
S. cerevisiae (*MATa his3Δ200 leu2Δ0 lys2Δ0 trp1Δ63 ura3Δ0 met15Δ0 hht1-hhf1::NatMX4 can1::MFA1pr-HIS3 hht2-hhf2::URA3-hhts(K14Q)-HHFS*)	Horizon Discovery

Table S2: Plasmids

plasmid	source
YEplac181	Gietz and Sugino (S2)
YEplac181-IGS	This manuscript

Table S3: Antibodies

antibody	source	identifier
Goat polyclonal anti-Sir2 (yN-19)	Santa Cruz Biotechnology	Cat# sc-6666; RRID: AB_656455
Donkey polyclonal anti-goat IgG-HRP	Santa Cruz Biotechnology	Cat# sc-2020; RRID: AB_63728
Rat monoclonal anti-tubulin alpha: HRP (clone YL1/2)	Bio-Rad	Cat# MCA77P; RRID: AB_2021090
Rabbit polyclonal anti-histone H3	Abcam	Cat# ab1791, RRID: AB_302613
Rabbit polyclonal anti-acetyl-histone H3 (Lys9)	Millipore	Cat# 07-352, RRID: AB_310544
Rabbit polyclonal anti-acetyl-histone H3 (Lys14)	Millipore	Cat# 07-353, RRID: AB_310545
Rabbit polyclonal anti-histone H4K16 Ac	Active Motif	Cat# 39167, RRID: AB_2636968
Mouse monoclonal anti-HA-tag (F-7) HRP	Santa Cruz Biotechnology	Cat# sc-7392 HRP RRID: AB_2894930
Rat monoclonal RNA pol II antibody (mAb)	Active Motif	Cat# 61081 RRID: AB_2793500

Table S4: Oligonucleotides

For Southern blot (probe 1)<br><br>CATTTCCTATAGTTAACAGGACATGCC
For Southern blot (probe 1)<br><br>AATTCGCACTATCCAGCTGCACTC
For Southern blot (probe 2)<br><br>GGCGAGGTTCAGAAAACTGTCG
For Southern blot (probe 2)<br><br>AAACGGCAAGAATGCGTTGTTTG
For qPCR (to detect E-pro transcription)<br><br>CCCATAACTAACCTACCATTCGA
For qPCR (to detect E-pro transcription)<br><br>TCAAGTAGTAGCAACCCAATGAG
For qPCR (to detect *ACT1* mRNA and *ACT1* region)<br><br>CGAATTGAGAGTTGCCCCAG
For qPCR (to detect *ACT1* mRNA and *ACT1* region)<br><br>CAAGGACAAAACGGCTTGGA

For qPCR (to detect E-pro region)<br><br>GCGGTATGCGGAGTTGTAAG
For qPCR (to detect E-pro region)<br><br>CGGTTTTGTTCTCTTCCCTCC
For qPCR (to detect *SIR2* mRNA)<br><br>CCGAGGATTTGAACTCGTTATAC
For qPCR (to detect *SIR2* mRNA)<br><br>CCAAATCTTGAACACGCTCTTGC
For qPCR (to detect *GCN5* mRNA)<br><br>CATCAGATTGAAGAGGATCACTTG
For qPCR (to detect *GCN5* mRNA)<br><br>GGTGCCCTCTTGTTTATTGGTCTC
For qPCR (to detect TEL07L)<br><br>AACCACCATCCATCTCTCTACTTACTACTA
For qPCR (to detect TEL07L)<br><br>AGAACAACAGTACAGTGAGTAGGACATG

Supplemental References

S1. Nogi,Y., Yano,R. and Nomura,M. (1991) Synthesis of large rRNAs by RNA polymerase II in mutants of Saccharomyces cerevisiae defective in RNA polymerase I. *Proc. Natl. Acad. Sci. U. S. A.*, **88**, 3962–3966.

S2. Gietz,R.D. and Sugino,A. (1988) New yeast-Escherichia coli shuttle vectors constructed with in vitro mutagenized yeast genes lacking six-base pair restriction sites. *Gene*, **74**, 527–534.
